# Evaluating the Predictive Performance of miR-124-2 and FAM19A4 for Cervical Lesions in a Single Center from Romania: A Prospective Study

**DOI:** 10.3390/jcm14103452

**Published:** 2025-05-15

**Authors:** Ioana-Sadiye Scripcariu, Tudor Gisca, Anca Botezatu, Demetra Socolov, Ingrid-Andrada Vasilache, Carmen Diaconu, Alina Fudulu

**Affiliations:** 1 Department of Mother and Child Care “Grigore T. Popa”, University of Medicine and Pharmacy, 700115 Iasi, Romania; isscripcariu@gmail.com (I.-S.S.);; 2 Stefan S. Nicolau Institute of Virology, Romanian Academy, 030304 Bucharest, Romania; ccdiaconu@yahoo.com (C.D.); alina.fudulu@virology.ro (A.F.)

**Keywords:** miR-124-2, FAM19A4, cervical neoplasia, human papillomavirus, methylation analysis

## Abstract

**Background/Objectives**: Molecular triage strategies for cervical lesions based on miR-124-2 and FAM19A4 are poorly studied in various populations. The aim of this prospective study was to evaluate the individual and combined predictive performance of these two markers for the prediction of various histological categories of cervical lesions. **Methods**: An FAM19A4 and miR124-2 methylation analysis was performed on 70 samples from patients negative for intraepithelial lesion or malignancy (NILM), cervical intraepithelial neoplasia (CIN)1-3 and squamous cervical carcinoma (SCC), along with human papillomavirus (HPV) genotyping and cytological and histopathological assessment. Descriptive statistics examined clinical associations, while sensitivity analysis evaluated the predictive performance of these markers individually and combined. **Results**: The sensitivity of miR-124-2 was 28.1%, while its specificity was 86.8% for SCC. The ROC values ranged between 0.25 and 0.62 for the evaluated histological categories, suggesting a poor to moderate predictive performance. FAM19A4 had a sensitivity of 36% for predicting CIN3 and SCC, as well as a high specificity for CIN3 and SCC (88.9%), with ROC values between 0.35 and 0.73 for the evaluated histological categories. The combined tests improved the PPV for higher-risk lesions (CIN3, SCC), but did not significantly improve the ROC values. FAM19A4 achieved the best performance for the prediction of CIN2+ (ROC: 0.64) and CIN3+ lesions (ROC: 0.73). **Conclusions**: We hypothesize that while not suitable as stand-alone diagnostic tools, such biomarkers may aid in stratifying patients and optimizing referral decisions, pending further validation in larger, population-based cohorts.

## 1. Introduction

Cervical cancer continues to be one of the most prevalent cancers among women globally. According to the latest statistics from 2022 (GLOBOCAN), nearly 661,021 new cases and almost 348,189 cervical cancer-related deaths were reported [1]. Moreover, cervical cancer continues to be a significant public health challenge in Romania, with one of the highest mortality rates in the European Union [2,3].

Despite the availability of the human papillomavirus (HPV) vaccine, which has proven effective in reducing the incidence of HPV-related cervical cancers, the need for effective screening remains crucial. Thus, screening programs are essential to detect precancerous changes and intervene before the disease becomes life-threatening [4].

Currently, the main screening method used to identify women with cervical lesions and to establish the risk for cervical cancer development is the Papanicolaou (Pap) test. Besides cytology-based triage, HPV detection and genotyping are needed to stratify women with clinical disease from women with transient HPV infections or low-risk HPV genotypes. HPV testing has improved the screening for cervical cancer prevention, as cytology can be a subjective method. New strategies for cervical screening are needed in order to reduce cervical cancer frequency [5].

In recent years, advancements in molecular biology have introduced more specific methods of cervical cancer screening, among which DNA methylation testing has emerged as a promising tool. Specifically, the methylation of HPV-related genes and tumor suppressor genes has been associated with the progression of cervical precancerous lesions into invasive cancer [6].

Methylation testing offers several advantages over traditional screening methods like the Pap test or HPV testing alone. It has the potential to improve sensitivity and specificity, identifying women at the highest risk of developing cervical cancer earlier and more accurately [7]. Such information is particularly valuable in countries like Romania, where late-stage diagnosis is still common due to barriers in healthcare access and participation in screening programs. Also, the methylation level of some markers could be influenced by HPV vaccination [8].

Among all the methylation biomarkers studied, some of them have proven clinically relevant for the diagnosis, prognosis and treatment of cervical cancer. These include CADM1, EPB41L3, FAM19A4, MAL, miR-124, PAX1, DPP6, RALYL, GSX1 and SOX1 [9,10]. The QIAsure Methylation Test is a recently clinically validated CE-IVD commercial triage assay that utilizes multiplex quantitative methylation-specific PCR (qMSP) to detect hypermethylation in two disease-related genes (FAM19A4 and hsa-mir124-2), along with beta-actin as a reference gene [11]. It was shown that persistent high-risk HPV infection induces host DNA damage, leading to the aberrant methylation of tumor suppressor genes such as FAM19A4 and miR-124-2, with the subsequent loss of tumor-suppressive function and progression to high-grade cervical intraepithelial neoplasia (CIN2/3) and invasive cancer.

As a triage test for HPV-positive women, FAM19A4/miR124-2 methylation analysis offers a clinical sensitivity and specificity comparable to cytology. Moreover, it has shown high intra-laboratory and inter-laboratory consistency across various cervical screening sample collection methods [12]. It was demonstrated that the FAM19A4/miR124-2 methylation assay detects 98.3% of all cervical cancers, regardless of histotype, HPV genotype, geographic region and sample type, as was shown by a study that involved 519 cervical cancers patients [13].

The methylation analysis of FAM19A4 and hsa-mir124-2 in HPV-positive exfoliated cervical cell specimens has proven to be a sensitive test for identifying women with cervical cancer and high-grade CIN who require treatment [14]. An international study based on a large cohort showed that the QIAsure Methylation Test is a highly reproducible assay and may be used to discern hrHPV-positive women with a clinically relevant cervical disease [12].

This study explored the potential of DNA methylation as a novel screening method for cervical cancer, emphasizing its ability to complement existing Pap and HPV tests. To our knowledge, this is the first prospective study conducted in Romania that intends to evaluate the clinical utility of the QIAsure Methylation Test as a triage tool for HPV-positive women. Specifically, we aimed to evaluate the predictive performance of miR124-2 and FAM19A4 methylation status for the prediction of cervical lesions, either individually or combined.

## 2. Materials and Methods

### 2.1. Study Design and Sample Collection

This study included cervical specimens that fitted the inclusion criteria from a total of 512 women who underwent evaluation at a private clinic in the northeast of Romania for targeted gynecological examinations between January 2024 and December 2024 (Ethical Approval No. 12/1 November 2023). The inclusion criteria for this study were as follows: women aged 18 and above, not currently pregnant. Specifically, individuals with known immunosuppression (e.g., HIV-positive status, post-transplant immunosuppression, or chronic immunosuppressive therapy) were excluded. Additionally, participants were required to have abstained from vaginal contact or showers for a minimum of three days prior to sampling. Prior to inclusion in this study, all participants were fully informed about the purpose and details of the research and provided their informed consent by signing a consent form.

The diagnosis, management and follow-up of the patients were in accordance with national guidelines and the World Health Organization (WHO) guidelines for the screening and treatment of cervical precancerous lesions for cervical cancer prevention [15]. The patients were invited to fill out a standardized questionnaire designed to gather information regarding their ethnicity, education, age, marital status, smoking and drinking habits, medical history, sexual activity and place of residence.

Cervical samples for the Pap test and HPV genotyping were collected using a cervix brush (Hologic, Bedford, MA, USA) and processed with the ThinPrep liquid-based method following the manufacturer’s instructions (ThinPrep-Hologic, Bedford, MA, USA). For DNA methylation analysis, samples were collected with an ESwab (COPAN, Brescia, Italy) and preserved at −80 °C, and all specimens were collected during a single visit.

Cytological diagnoses were made following the grading criteria of the Bethesda System [16], which includes the following categories: Negative for Intraepithelial Lesion or Malignancy (NILM); Atypical Squamous Cells of Undetermined Significance (ASCUS), Atypical Squamous Cells That Cannot Exclude High-Grade Squamous Intraepithelial Lesion (ASCH); Low-Grade Squamous Intraepithelial Lesion (LGSIL); High-Grade Squamous Intraepithelial Lesion (HGSIL); and Squamous Cell Carcinoma (SCC).

The management of patients based on the cytology and HPV tests was as follows:

- women who tested HPV-negative and had normal cytology were referred to routine screening (screening interval 5 years);
- HPV-positive women with normal cytology or with ASCUS or low-grade LSIL were retested for both HPV and cytology at 6 and 18 months and referred for colposcopy in case of persistence or worsening of abnormal Pap test;
- women with HSIL or worse were directly referred for colposcopy.

**Figure 1:**
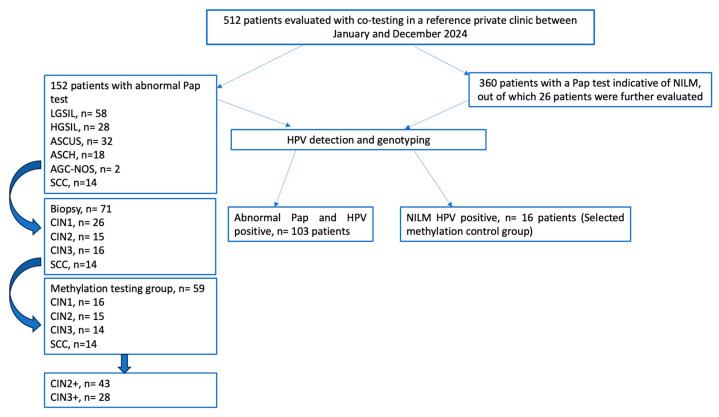
Study flowchart—cervical cancer screening pathway for clinician-collected samples.

Based on these management criteria, a total of 30 patients underwent colposcopy and punch biopsy (PB), 10 patients underwent cone biopsy and a total of 31 needed a Large Loop Excision of the Transformation Zone (LLETZ). The histology results were classified as CIN grade 1, 2 or 3 or SCC according to the Richart classification [17] (Figure 1). According to the biopsy results, 26 patients exhibited CIN1 lesions, 15 patients exhibited CIN2 lesions, 14 patients exhibited CIN3 lesions and 14 patients exhibited SCC. From this sample, we identified a total of 59 patients with adequate sample material for methylation testing: CIN1 (16 patients), CIN2 (15 patients), CIN3 (14 patients) and SCC (14 patients).

Only those who underwent a colposcopic biopsy and had sufficient high-quality biological material suitable for molecular analysis were included in the final cohort. This resulted in a total of 70 patients (control group *n* = 16–22.85%; investigated group *n* = 54–77.15%) who were tested using the QIAsure Methylation Test.

### 2.2. DNA Isolation

DNA was isolated from cervical samples using High Pure QIAamp DNA Mini Kit (Qiagen, Valencia, CA, USA) according to manufacturer’s instructions. Concentration and purity of each DNA sample were determined using NanoDrop ND1000 spectrophotometer (Thermo Fisher Scientific Inc., Waltham, MA, USA).

### 2.3. HPV Detection and Genotyping

Human papillomavirus detection and genotyping was performed for all samples using Allplex™ HPV28 Detection (Seegene Technologies Inc. Europe, Dusseldorf, Germany) according to the manufacturer’s instructions. The kit is a multiplex real-time PCR assay that allow the simultaneous amplification and detection of the target nucleic acids of 19 high-risk HPV types (hrHPV) (16, 18, 26, 31, 33, 35, 39, 45, 51, 52, 53, 56, 58, 59, 66, 68, 69, 73, 82) and 9 low-risk HPV (lr-HPV) types (6, 11, 40, 42, 43, 44, 54, 61, 70), as well as an internal control (IC).

### 2.4. FAM19A4 and miR124-2 Methylation Analysis

In order to evaluate FAM19A4 and miR124-2 using a gene methylation analysis, a DNA bisulfite treatment was performed using an EpiTect Fast Bisulfite kit (Qiagen, Valencia, CA, USA). A total of 200 ng/µL of each isolated DNA sample was treated for cytosine conversion. After treatment, the DNA samples were stored at −20 °C until use.

FAM19A4 and miR124-2 methylation analysis was performed using bisulfite-converted DNA from the cervical samples by quantitative methylation-specific PCR (qMSP) with a QIAsure Methylation Test^®^ IVD (Qiagen, Hilden, Germany).

As an input, the assay uses 2.5 µL of bisulfite-converted DNA and qMSP is performed on a Rotor-Gene Q MDx 5plex HRM instrument. Amplification, data analysis and reporting are controlled by Rotor-Gene AssayManager v1.0 software (QIAGEN GmBH, Hilden, Germany), which utilizes a fixed assay profile. The analysis software includes quality control steps and standardized cutoff thresholds, as defined by the manufacturer.

The test includes an internal control (ACTB) for DNA integrity and PCR performance evaluation, along with a non-template control (NTC) to verify any possible contamination. If all of the controls in the run are valid, then the Rotor-Gene AssayManager v1.0 will analyze the patient’s samples. The CT value of the housekeeping gene ACTB must be ≤26.4 for each sample in order to be validated by the Rotor-Gene AssayManager.

### 2.5. Statistical Analysis

The categorical variables were compared between groups with chi-squared or Fisher’s exact tests and were presented as frequencies with corresponding percentages. We also performed a sensitivity analysis for determining the individual and combined predictive performance of these two markers for the prediction of various histological categories of cervical lesions. We expanded the histopathological categories to CIN2+ (43 patients) and CIN3+ (28 patients), also testing their predicting performance.

We determined the sensitivity, specificity, positive predictive value (PPV), negative predictive value (NPV), positive likelihood ratio (LR+), negative likelihood ratio (LR−) and area under the receiver operating characteristic curve (ROC) values. We also performed multivariable logistic regressions of individual and combined markers for the prediction of histological categories, which were adjusted for age, and we reported the results as odds ratios (ORs) and 95% confidence intervals (CIs). The statistical analyses were performed using STATA SE software (version 18.5, released in 2024. StataCorp LLC., Lakeway Dr., College Station, TX, USA).

## 3. Results

From the 178 women who met the criteria to be included in the study, 70 patients were selected to be further analyzed for miR124-2 and FAM19A4 methylation status. The group of patients was assessed in a balanced manner as follows: control group—NILM (*n* = 13), CIN1 (*n* = 14), CIN2 (*n* = 15), CIN3 (*n* = 14) and SCC (*n* = 14). The median age of the included patients was 38 years (range 25–87 years).

The data collected from the patients’ questionnaires were analyzed regarding their histological groups. The results showed that the diagnosis of cancer in the patients was significantly more frequent in individuals over 45 years old. Additionally, the patients diagnosed with cervical cancer were from rural areas. The majority (85.71%) of cancer patients had only one partner. Although the number of pregnancies and births was higher in the group of cancer patients, this did not appear to significantly influence the outcome (Table 1).

Pap smears revealed abnormalities including LGSIL in 18 women (26%), ASCUS in 8 (11%), ASC-H in 5 (7%), HGSIL in 11 (16%) and SCC in 12 (17%) women. The cytological diagnosis was confirmed by histology, particularly in patients diagnosed with LGSIL, HGSIL and SCC. The ASCUS and ASCH groups were more heterogeneous, with histological diagnoses revealing varying neoplasia grades. In the ASCUS group, 50% of patients had CIN1, and 25% each had CIN2 and CIN3, while in the ASCH group, 40% presented with CIN2 and 60% with CIN3. A total of 12 patients had a prior diagnosis of ASCUS, while 10 patients had a history of LSIL, 8 patients had a history of HSIL and 4 patients were documented with SCC. In terms of diagnostic interventions, 15 patients had undergone cervical biopsies, and 10 patients had a history of excisional procedures such as conization or LLETZ.

Cytology significantly correlated with both age (*p* < 0.0001) and rural living (*p* = 0.0299), as well as the frequency of sexual intercourse (*p* < 0.0001), with results being similar to those obtained based on the degree of neoplasia.

In this study, 52/70 samples (74.29%) were positive for at least one HR-HPV genotype. Almost all CIN3 and SCC samples (92.86% each) were positive for HPV infection, and a higher percentage of positive samples was also observed in the CIN2 patients (80%), as shown in Table 2. The number of patients positive for HPV infection increased with the severity of the lesion. Most of the patients presented with a single-genotype infection (41%), 33% had multiple infections and 26% were negative.

Infections with multiple genotypes were found in low-grade lesions, and significant differences were observed (*p* = 0.0047). Single-genotype infections being more common in CIN3 and SCC cases (57.14% for both), whereas co-infections were more frequent in CIN2 cases (53.33%).

**Figure 2:**
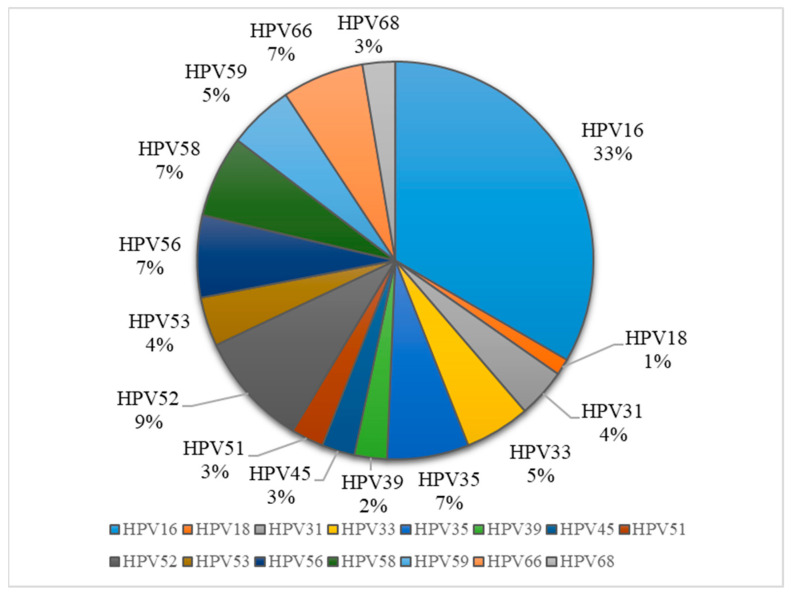
Distribution of HPV genotypes in sample collection.

The most frequent genotype was HPV16 (33%), followed by HPV52 (9%) and equally (7%) HPV35, 56, 58 and 66 (Figure 2). The frequency of the HPV16 strain increased with cervical lesion severity: 28.57% of CIN1 (4/14), 40% of CIN2 (6/15), 64.29% of CIN3 (9/14) and 35.71% of carcinoma (5/14). Regarding HPV16 infection, we observed that 12/52 (23.08%) were with a single infection, while 14/52 (26.92%) presented multiple infections. The most common combinations involved HPV16 co-infections with HPV52, HPV59 and HPV39, each identified in two cases. Less frequent combinations included HPV16 with genotypes such as HPV33, HPV58, HPV66, HPV61, HPV53, HPV82 and HPV56 (each observed in one case). Additionally, we detected a co-infection involving HPV16, HPV33 and HPV58 in one case, as well as a case with multiple co-infections comprising HPV16, HPV52, HPV56, HPV59, HPV66 and HPV82.

**Figure 3:**
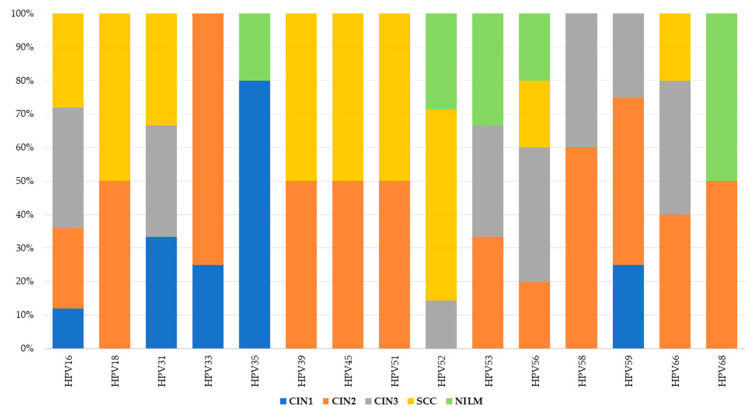
HPV genotype distributions in sample collection across histological categories.

The most common genotype in patients with dysplasia and cancer was HPV16. In the case of CIN2 and CIN3, there was a higher frequency of HPV58 and HPV56 genotypes. Notably, HPV33 was also observed in CIN2, while HPV35 was found in CIN1. In cancer cases, alongside HPV16, there was an increased number of infections with HPV52. HPV68 was present in equal proportions across all study groups. Interestingly, cases with NILM cytology showed a higher frequency in the HPV52 genotype (Figure 3).

The Qiasure test results were first evaluated in relation to the histological categories. As observed in Table 2, the methylation of the miR124-2 and FAM19A4 genes increases gradually from CIN1 to SCC. The methylation of these two genes presented a similar pattern, increasing with the neoplasia grade and having identical results in cervical carcinoma samples. Each histological category included one case that was hrHPV-negative but positive for at least one methylation marker. While the CIN1 and CIN2 cases showed miR-124-2 methylation and the SCC case exhibited FAM19A4 methylation, the CIN3 case displayed methylation in both of the investigated markers. Notably, miR-124-2 gene methylation was significantly more prevalent in the NILM samples compared to FAM19A4 gene methylation.

We further investigated the correlation between HPV infection status and the methylation test results in relation to cytology. Almost all patients diagnosed with cancer were HPV-positive, and the same was true for those with HGSIL. The occurrence of multiple infections decreased with the progression of the lesion, the results being similar to those obtained after the histopathological examination. Also, the degree of methylation for miR-124-2 and FAM19A4 increased significantly (*p* = 0.0043 and 0.0051, respectively) with the progression of the lesion (Table 3).

HPV infection did not appear to influence the methylation status of both tested genes (Table 4). However, for both miR-124-2 and FAM19A4, methylation was positive in single-HPV-genotype infections at a higher percentage (40.62% and 48%, respectively) than in multiple infections.

A sensitivity analysis of individual markers and a combined approach for the prediction of histological categories is presented in Table 5. Our results indicated that miR-124-2 best predicted SCC; however, the test sensitivity was low (28.1%) and the specificity was high (86.8%). The ROC values ranged between 0.25 and 0.62, suggesting a poor to moderate predictive performance.

On the other hand, FAM19A4 best predicted high-grade lesions such as CIN3 and SCC (Sensitivity: 36.0%). It also presented high specificity for the prediction of CIN3 and SCC (88.9%). Its ROC values between 0.35 and 0.73 indicate a low to moderate performance for the evaluated categories.

The combined tests improved the PPV for higher-risk lesions (CIN3, SCC), but did not significantly improve the ROC values. The sensitivity for CIN3 was 28.9%, higher than miR-124-2, but lower than FAM19A4. The ROC values ranged between 0.34 and 0.67, indicating a low to moderate predictive performance for the evaluated histological categories.

We further expanded the sensitivity analysis of individual and combined markers for the prediction of the CIN2+ and CIN3+ histological categories. Our results indicated that FAM19A4 had the highest sensitivity for detecting CIN2+ (80%) and CIN3+ (72%), as well as the highest NPV for CIN2+ (81.5%) and for CIN3+ (83.3%), compared to other approaches. Furthermore, its corresponding ROC values (0.64 and 0.73) suggest a moderate predictive accuracy for CIN3+ lesions.

Compared to FAM19A4, miR-124-2 achieved a lower sensitivity (65.6% for CIN2+, 53.1% for CIN3+), a lower specificity (42.1% for CIN2+ and 71.1% for CIN3+) and lower ROC values (0.54 for CIN2+, 0.62 for CIN3+).

The combined approach improved the PPV (60.5% for CIN2+ and 75% for CIN3+), making it more useful in confirming CIN3+ cases, but it did not significantly improve the sensitivity. Its corresponding ROC values (0.58 and 0.67) suggest a moderate predictive accuracy for CIN2+/CIN3+ lesions.

**Figure 4:**
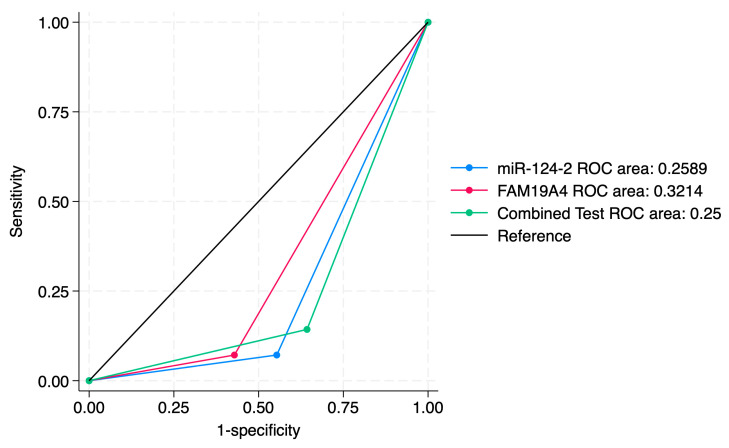
ROC comparisons between individual and combined approach for prediction of CIN1 lesions.

**Figure 5:**
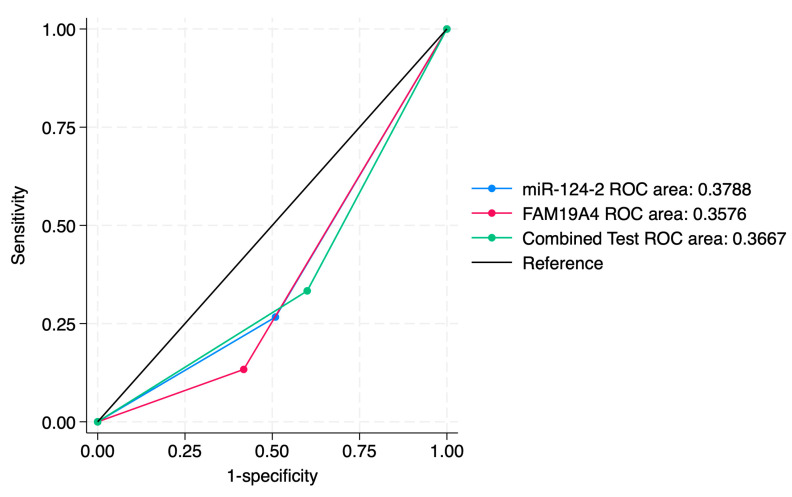
ROC comparisons between individual and combined approach for prediction of CIN2 lesions.

**Figure 6:**
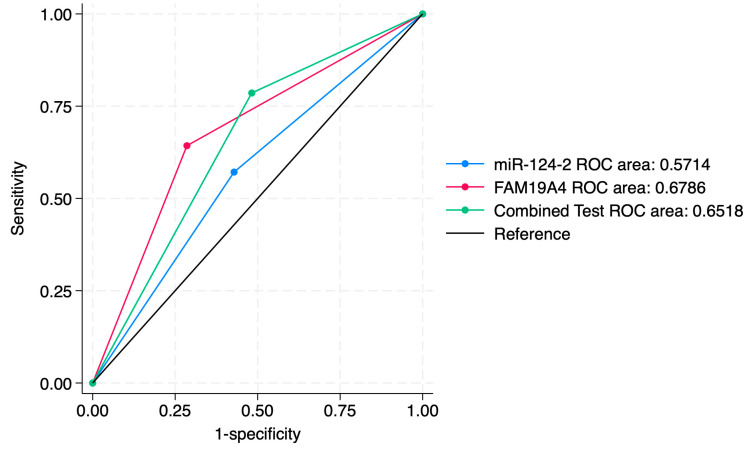
ROC comparisons between individual and combined approach for prediction of CIN3 lesions.

**Figure 7:**
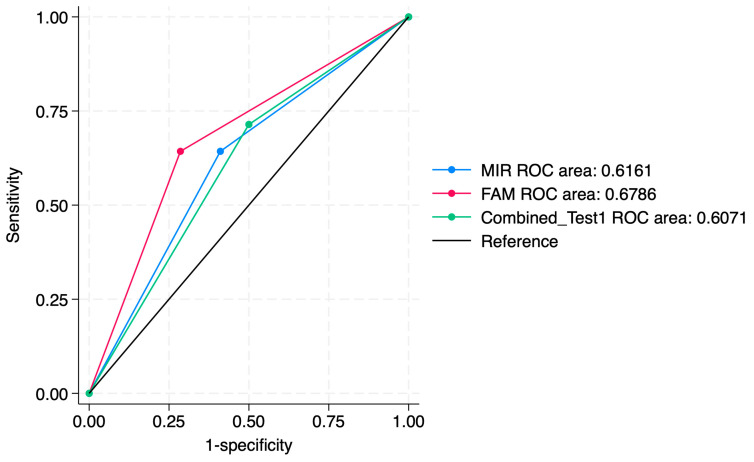
ROC comparisons between individual and combined approach for prediction of SCC.

**Figure 8:**
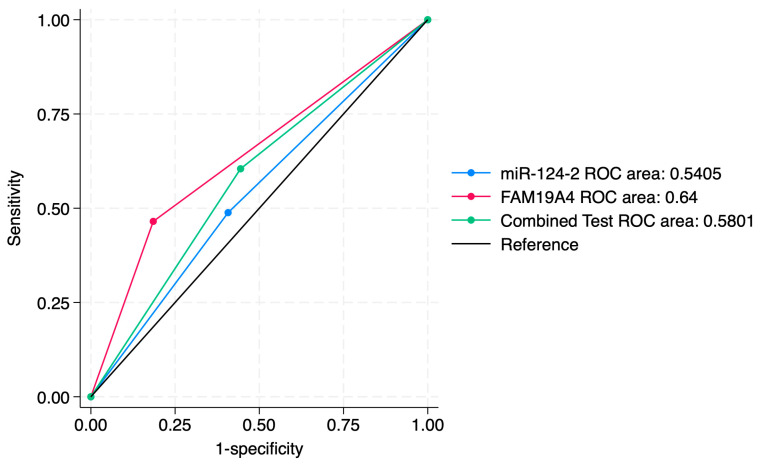
ROC comparisons between individual and combined approach for prediction of CIN2+.

**Figure 9:**
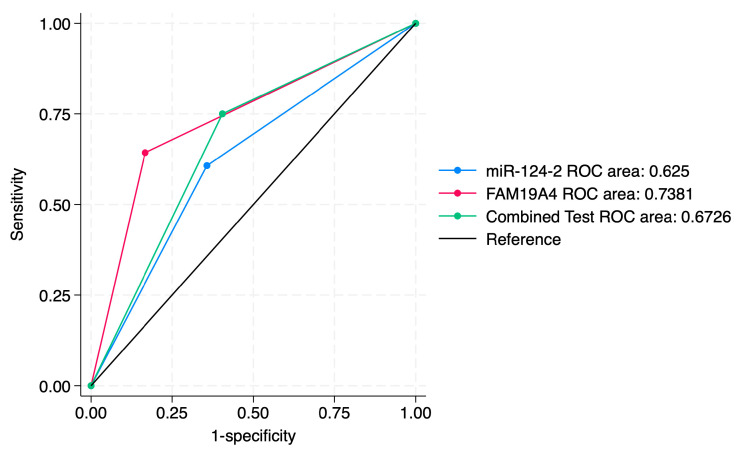
ROC comparisons between individual and combined approach for prediction of CIN3+.

ROC curve comparisons between the individual markers and the combined approach are presented in Figure 4, Figure 5, Figure 6, Figure 7, Figure 8 and Figure 9. In terms of ROC values, both miR-124-2 and FAM19A4 best predicted CIN3 lesions and SCC. The ROC values corresponding to the individual tests or combined approach did not indicate a satisfactory predictive performance (ROC range: 0.25–0.37). FAM19A4 achieved the best performance for the prediction of CIN2+ (ROC: 0.64) and CIN3+ lesions (ROC: 0.73).

The results of the multivariable logistic regressions of individual and combined markers for the prediction of histological categories are presented in Table 6. Our data indicated that FAM19A4 methylation emerged as a significant predictor of SCC (OR = 4.50; *p* = 0.017) and that after adjusting for age, the association remained statistically significant (OR = 8.28; *p* = 0.041). On the other hand, miR-124-2 did not significantly predict SCC before or after age adjustment.

FAM19A4 methylation was strongly associated with increased odds of CIN3 lesions (OR = 4.50; 95% CI: 1.31–15.51; *p* = 0.017), and this association remained significant after adjusting for age (OR = 5.51; *p* = 0.013). In contrast, miR-124-2 alone did not significantly predict CIN3 (OR = 1.78; *p* = 0.341), and remained non-significant even after adjusting for age.

None of the individual markers reached conventional statistical significance for CIN2 prediction. On the other hand, miR-124-2 methylation was significantly associated with a lower likelihood of CIN1 lesions (OR = 0.07; 95% CI: 0.0085–0.57; *p* = 0.013), suggesting an inverse relationship. This finding was also applicable for FAM19A4 methylation (OR = 0.11; 95% CI: 0.014–0.94; *p* = 0.044) and for the combined test (OR = 0.11; *p* = 0.006).

## 4. Discussion

While cytology was historically the primary screening method in Romania, current protocols recommend dual screening with cytology and HR-HPV genotyping for individuals aged 25–65 years. Accordingly, both methods were applied simultaneously in all patients included in our study [16].

For women with cytological abnormalities, HPV detection and genotyping are recommended. However, these methods sometimes cannot distinguish between transient, clinically insignificant HPV infections and persistent ones that require intervention. This limitation can lead to unnecessary follow-up visits, excessive colposcopy referrals and treatments, incurring substantial material, human and psychological costs. These methods have reached their full potential, highlighting the need for a new screening approach to better perform triage on patients at risk of developing cervical cancer. Molecular biomarkers are beginning to be integrated into clinical diagnostics and DNA methylation testing represents a promising advancement for cervical cancer screening [18].

An important aspect is the clinical management of low-grade cervical abnormalities, especially ASCUS and ASC-H, in cytology, as well as CIN1 from a histological perspective. Molecular tests that detect gene-specific methylation can be valuable tools not only for distinguishing between heterogeneous cytological lesions but also for predicting the outcomes of low- and intermediate-grade neoplasia stages (CIN1 and CIN2).

Our results indicated that the frequency of the HPV16 strain increased with cervical lesion severity: 28.57% of CIN1, 40% of CIN2, 64.29% of CIN3 and 35.71% of carcinoma cases. This observation is complementary to the findings of Plesa et al., who evaluated the HPV16 distribution in the Moldavian region of Romania [19]. The authors reported that HPV 16 was present as a single infection in 21.7% of CIN1 cases, 40.8% of CIN2 cases, 64.2% of CIN3 cases and 69% of SCC cases. Another study conducted in Romania indicated that the most frequent high-risk genotype was HPV16 (32.6%), followed by HPV18, HPV31 and HPV51 [20]. These observations suggest that while HPV16 is still the dominant genotype in our region, in the last 15 years, the incidence of HPV18 significantly decreased.

The present study showed that single-genotype infections were more common in CIN3 and SCC cases, while co-infections were found especially in CIN2 cases. This observation was highlighted by another study, which mentioned that multiple infections are more frequent among young women at the peak of their sexual activity or with an impaired immune response [21]. Bruno et al. showed that the prevalence of multiple infections is high in negative cases, cases of low-grade dysplasia and CIN2, decreasing in CIN3 until absent in neoplasia [22]. The same study indicated that single HPV infections were lower among negatives and CIN1, being more frequent in severe dysplasia and carcinoma. As lesions progress, immune evasion by the most oncogenic type (e.g., HPV16) may lead to the dominance of a single persistent infection in high-grade disease [23].

In this study, using a prospective approach, we evaluated the methylation status of FAM19A4 and hsa-miR124-2 in a subset of cervical samples across various neoplasia grades and squamous cell carcinoma (SCC), comparing these with a control group in the context of HPV infections. Similar studies have been conducted on large specimen cohorts across several European countries [24,25,26,27,28], as well as smaller cohorts [29,30]. However, even if it is a small cohort study, the present work is the first of its kind in Eastern Europe that analyzes the diagnostic accuracy of these two biomarkers individually and combined.

The results showed an increase in the presence of methylation with the progression of lesion severity, both from cytological analysis and histopathological characterization. The methylation of both biomarkers demonstrated the best sensitivity in identifying CIN3 lesions and cancer, although it was low. FAM19A4 proved to be a more specific biomarker for CIN1-3 lesions and SCC screening compared to miR-124-2, while miR-124-2 appears to be frequently methylated even in patients with normal cytology (NILM).

Our results regarding our Qiasure Methylation Test sensitivity for CIN2 (68.4%) and CIN3 (55.3%) are comparable with those reported in a multicenter study conducted in Scotland, Denmark, Slovenia and the Netherlands, where the overall sensitivity of the FAM19A4/miR124-2 methylation test was 46.8% (range: 33.3–61.1%) for CIN2 and 77.2% (range: 75–78.2%) for CIN3 [25]. However, this limitation was offset by a notable increase in specificity for high-grade lesions [25].

Moreover, a key aspect of this study was the identification of CIN and SCC cases that were hrHPV-negative but exhibited methylation of one of the two investigated factors, a finding also reported in a study with a larger cohort [13]. Since our findings indicated that some cases presented FAM19A4/miR124-2 methylation positivity and hrHPV-negativity, integrating this methylation assay with HPV screening could effectively rule out prevalent cancer and other lesions with high confidence.

The results obtained are similar to those published by Dippmann et al., who reported a positive predictive value of 51.4%, while in this study, it was 61%. In terms of the negative predictive value, it was lower compared to the aforementioned study—52.5% vs. 88.1% [31].

In our study, NILM cases that presented both genes’ methylation were positive for HPV infection with the HPV52 genotype, similar to SCC. In hrHPV-negative NILM cases, this may reflect persistent molecular changes despite a negative cytological test. De Strooper et al. found that a significant percentage of cancer cases had methylated cytology samples years before diagnosis, with 64% showing methylation 5–9 years prior and 33% at 10–14 years. This suggests that methylation can be detected early in cancer development, even when cytology results appear normal [29].

Vink et al. assessed FAM19A4/miR124-2 methylation in a large, global series of cervical cancer cases, revealing a positivity rate exceeding 98%. This suggests that the FAM19A4/miR124-2 methylation assay offers high sensitivity for detecting cervical cancer, and a negative result may exclude the presence of the disease [13].

In a study conducted by Dick et al., FAM19A4/miR124-2 methylation, HPV16/18 genotyping, HPV16/18/31/33/45 genotyping and their combinations were assessed in HPV-positive women with borderline or mild dyskaryosis (BMD) cytology using data from two large Dutch population-based screening cohorts [32]. The authors concluded that further risk stratification of HPV-positive women using FAM19A4/miR124-2 methylation testing could reduce direct colposcopy referrals by 60%, while maintaining a high sensitivity for detecting CIN3+.

Also, Dippman et al. performed an FAM19A4/miR124-2 methylation assay on a cohort of 195 cervical samples, including 46 normal cytology (NILM) and hrHPV-negative specimens [31]. As the assay is intended as a triage option for hrHPV-positive subgroups, the sensitivity and specificity measurements were specifically estimated on this group. Notably, their results indicated a sensitivity of 63.3% for CIN2+ and 78.6% for CIN3+, while the specificity was similar for both studied groups, 67.4% and 68.2%, respectively. The performance of the assay in hrHPV-negative NILM cases was particularly noteworthy, with around 23.9% of cases being positive.

The findings of Kremer et al.’s study demonstrated that a negative FAM19A4/miR124-2 methylation test could effectively identify women with CIN2/3 who were most likely to experience clinical regression. Moreover, clinical regression was found to be significantly linked to methylation status and HPV16 genotyping, regardless of whether the analysis was performed on clinician-collected or self-collected samples [28].

In our multivariable logistic regression analysis, FAM19A4 methylation emerged as a significant predictor of SCC and CIN3, even after adjusting for the patients’ age, suggesting a strong and independent predictive value. Verhoef et al. showed that FAM19A4/miR124-2 methylation achieved a positive predictive value of 32.1% and a negative predictive value of 92.2% for CIN3 detection, and these values were improved when they were associated with HPV genotyping [33].

In contrast, miR-124-2 methylation did not show a statistically significant association with SCC or CIN3 before or after adjusting for age. An observational study, however, indicated that miR124-2 methylation levels were significantly higher with an increasing grade of disease, and they were a significant predictor of HSIL [34].

Also, for CIN2, neither individual marker reached statistical significance, while both miR-124-2 and FAM19A4 methylations were significantly associated with a reduced likelihood of CIN1 lesions. A retrospective multicenter study indicated that a negative FAM19A4/miR124-2 methylation test can exclude progressive CIN disease in pregnant patients who had been previously diagnosed with CIN3 [35].

Why is the methylation test positive in NILM? The high presence of methylation in both genes in NILM coincides in our study with the high number of HPV52 cases, similar to SCC. It is possible that the methylation of the miR-124-2 gene is more sensitive to the presence of HPV52. In hrHPV-negative NILM cases that are positive for this test, it may reflect non-specific background methylation. Another hypothesis could be related to a persistence of molecular changes, even if the cytological test is negative.

In the current screening pathway, high-risk HPV (hrHPV)-positive samples are triaged using liquid-based cytology, with abnormal results referred for colposcopy and the potential treatment of CIN2+. However, liquid-based cytology has limitations: it is resource-intensive and suffers from declining specialist availability, variable performance and low specificity. These all lead to unnecessary interventions. Moreover, liquid-based cytology requires clinician-collected samples, adding extra appointments for self-sampled individuals and increasing costs and loss to follow-up, especially as self-sampling becomes more common. According to the results of a study conducted in the Netherlands, in an HPV primary screening program with self-sampling, triage with the QIAsure Methylation Test reduced the cost per screen compared to liquid-based cytology without creating a financial barrier to its implementation in the Dutch cervical cancer screening program [36]. Additionally, DNA methylation testing may improve laboratory workflow, enhance the patient experience and reduce loss to follow-up among HPV self-sampling users.

One limitation of our study is its small sample size, which restricts the generalizability of our results. Our study highlighted a relatively low sensitivity for both methylation markers when considered individually—particularly for miR-124-2. However, this limitation was offset by a notable increase in specificity for high-grade lesions. Importantly, when stratified by lesion severity, both miR-124-2 and FAM19A4 demonstrated their highest predictive performance for CIN3 and SCC, as reflected by their respective ROC curve values. This suggests that while these markers may not be ideal for early lesion detection in isolation, together with other biomarkers or clinical information, they may hold clinical value in triaging women at greatest risk for developing high-grade lesions.

The clinical and diagnostic accuracy of methylation tests must be confirmed through their inclusion in larger prospective studies and by considering the essential role of various epidemiological and clinical variables.

## 5. Conclusions

With the growing availability of IVD-certified methylation testing kits, further research is needed to identify the most predictive test for oncogenesis and HPV infection. Standardizing methylation evaluation for screening requires international validation guidelines, reliable target genes, technical specifications and strategies for managing invalid results.

Our results demonstrated that while miR-124-2 methylation exhibited limited sensitivity across most histologic categories, it showed the highest specificity for detecting SCC, with a moderate predictive value. The same tendency was observed for FAM19A4 methylation when used to predict CIN3 lesions and SCC.

These sensitivities and negative predictive values were improved when they were used to predict CIN2+ and CIN3+ lesions, but their overall predictive accuracy remained moderate. On the other hand, using combined tests improved the PPV for higher-risk lesions (CIN3, SCC), but did not significantly improve the ROC values.

We hypothesize that while not suitable as stand-alone diagnostic tools, such biomarkers may aid in stratifying patients and optimizing referral decisions, pending further validation in larger, population-based cohorts.

## Figures and Tables

**Table 1:** Demographic characteristics and histology results of included samples.

Histology	NILM	CIN1	CIN2	CIN3	SCC	
*Number of cases*	*n* = 16	*n* = 16	*n* = 15	*n* = 14	*n* = 14	*p*-value
(% from total number of cases)	(18.57%)	(20%)	(21.43%)	(20%)	(20%)	
*Age*						
25–34 years old	6 (46.15%)	4 (28.57%)	6 (40%)	8 (57.14%)	1 (7.14%)	0.0031
35–44 years old	4 (30.77%)	9 (64.29%)	6 (40%)	4 (28.57%)	3 (21.43%)	
45+ years old	3 (23.08%)	1 (7.14%)	3 (20%)	2 (14.29%)	10 (71.43%)	
*Smoking*						
Yes	3 (23.08%)	3 (21.43%)	4 (26.67%)	5 (35.71%)	2 (14.29%)	0.5971
No	10 (76.92%)	11 (78.57%)	11 (73.33%)	9 (64.29%)	12 (85.71%)	
*Education level*						
Elementary school	2 (15.38%)	0	0	0	2 (14.29%)	0.0679
Professional school	1 (7.69%)	3 (21.43%)	2 (13.33%)	4 (28.57%)	6 (42.86%)	
High school	2 (15.38%)	6 (42.86%)	8 (53.33%)	6 (42.86%)	5 (35.71%)	
Bachelor	8 (61.54%)	5 (35.71%)	5 (33.33%)	4 (28.57%)	1 (7.14%)	
*Living area*						
Urban	12 (92.31%)	9 (64.29%)	14 (93.33%)	11 (78.57%)	5 (35.71%)	0.0028
Rural	1 (7.69%)	5 (35.71%)	1 (6.67%)	3 (21.43%)	9 (64.29%)	
*Marital status*						
Married	12 (92.31%)	10 (71.43%)	10 (66.67%)	9 (64.29%)	7 (50%)	<0.0001
Not married	1 (7.69%)	4 (28.57%)	5 (33.33%)	5 (35.71%)	0	
Widowed	0	0	0	0	7 (50%)	
*Age of first sexual experience*						
<20 years old	6 (46.15%)	8 (57.14%)	8 (53.33%)	9 (64.29%)	10 (71.43%)	0.7083
≥20 years old	7 (53.85%)	6 (42.86%)	7 (46.77%)	5 (35.71%)	4 (28.57%)	
*Number of sexual partners*						
1	6 (46.15%)	10 (71.43%)	5 (33.33%)	3 (21.43%)	12 (85.71%)	0.0104
2	4 (30.77%)	0	3 (20%)	4 (28.57%)	2 (14.29%)	
≥3	3 (23.08%)	4 (28.57%)	7 (46.67%)	7 (50%)	0	
*Sexual frequency*						
1 time per week	2 (15.38%)	9 (64.29%)	6 (40%)	3 (21.43%)	1 (7.14%)	<0.0001
2–3 times a week	10 (76.92%)	5 (35.71%)	9 (60%)	8 (57.14%)	0	
≥4 times a week	1 (7.69%)	0	0	3 (21.43%)	1 (7.14%)	
No	0	0	0	0	12 (85.71%)	
*Pregnancies*						
≤1	4 (30.77%)	5 (35.71%)	8 (53.33%)	9 (64.29%)	4 (28.57%)	0.2370
≥2	9 (69.23%)	9 (64.29%)	7 (46.77%)	5 (35.71%)	10 (71.43%)	
*Abortions*						
≤1	11 (84.62%)	10 (71.43%)	14 (93.33%)	13 (92.86%)	12 (85.71%)	0.4568
≥2	2 (15.38%)	4 (28.57%)	1 (6.67%)	1 (7.14%)	2 (14.29%)	
*Births*						
≤1	4 (30.77%)	6 (42.86%)	8 (53.33%)	10 (71.43%)	5 (35.71%)	0.2162
≥2	9 (69.23%)	8 (57.14%)	7 (46.77%)	4 (28.57%)	9 (64.29%)	

Legend: NILM—negative for intraepithelial lesion or malignancy; CIN1,2,3—cervical intraepithelial neoplasia 1,2,3; SCC—squamous cell carcinoma.

**Table 2:** Association between FAM19A4/miR124-2 methylation test results and histological categories.

Histology	NILM	CIN1	CIN2	CIN3	SCC	
*Number of cases*	*n* = 13	*n* = 14	*n* = 15	*n* = 14	*n* = 14	*p*-value
(% from total number of cases)	(18.57%)	(20%)	(21.43%)	(20%)	(20%)	
*HPV status*						0.0011
Positive	4 (30.77%)	10 (71.43%)	12 (80%)	13 (92.86%)	13 (92.86%)	
Negative	9 (69.23%)	4 (28.57%)	3 (20%)	1 (7.14%)	1 (7.14%)	
*HPV infection*						0.0047
Single infection	2 (15.38%)	7 (50%)	4 (26.67%)	8 (57.14%)	8 (57.14%)	
Multiple infections	2 (15.38%)	3 (21.43%)	8 (53.33%)	5 (35.71%)	5 (35.71%)	
No infection	9 (69.23%)	4 (28.57%)	3 (20%)	1 (7.14%)	1 (7.14%)	
*miR-124-2*						0.0010
Positive	10 (76.92%)	1 (7.14%)	4 (26.67%)	8 (57.14%)	9 (64.29%)	
Negative	3 (23.08%)	13 (92.86%)	11 (73.33%)	6 (42.86%)	5 (35.71%)	
*FAM19A4*						0.0007
Positive	4 (23.08%)	1 (7.14%)	2 (13.33%)	9 (64.29%)	9 (64.29%)	
Negative	9 (76.92%)	13 (92.86%)	13 (86.67%)	5 (35.71%)	5 (35.71%)	

Legend: HPV—human papillomavirus; NILM—negative for intraepithelial lesion or malignancy; CIN1,2,3—cervical intraepithelial neoplasia 1,2,3; SCC—squamous cell carcinoma.

**Table 3:** Association between FAM19A4/miR124-2 methylation test results and cytological categories.

Cytology	NILM	LGSIL	ASCUS	HGSIL	ASCH	SCC	*p*-Value
*HPV status*							0.0004
Positive	5 (31.25%)	14 (77.8%)	8 (100%)	9 (81.82%)	5 (100%)	11 (91.67%)	
Negative	11 (68.75%)	4 (22.2%)	0	2 (18.18%)	0	1 (8.43%)	
*HPV infection*							0.0014
Single infection	3 (18.75%)	6 (33.33%)	5 (62.5%)	3 (27.27%)	4 (80%)	8 (66.67%)	
Multiple infections	2 (12.5%)	8 (44.45%)	3 (37.5%)	6 (54.54%)	1 (20%)	3 (25%)	
No infection	11 (68.75%)	4 (22.22%)	0	2 (18.18%)	0	1 (8.33%)	
*miR-124-2*							0.0043
Positive	11 (68.75%)	2 (11.11%)	2 (25%)	7 (63.64%)	2 (40%)	8 (66.67%)	
Negative	5 (31.25%)	16 (88.89%)	6 (75%)	4 (36.36%)	3 (60%)	4 (33.33%)	
*FAM19A4*							0.0051
Positive	4 (25%)	1 (5.55%)	3 (37.5%)	7 (63.64%)	2 (40%)	8 (66.67%)	
Negative	12 (75%)	17 (94.45%)	5 (62.5%)	4 (36.36%)	3 (60%)	4 (33.33%)	

Legend: HPV—human papillomavirus; NILM—negative for intraepithelial lesion or malignancy; CIN1,2,3—cervical intraepithelial neoplasia 1,2,3; SCC—squamous cell carcinoma.

**Table 4:** Association between miR-124-2, FAM19A4 methylation status and HPV infection.

Number of Cases	HPV Status	HPV Infection	FAM19A4
(% from total number of cases)	Positive Negative	Single infection Multiple infections No infection	Positive Negative
*miR-124-2 positive* *n* = 32 (45.71%)	23 (71.87%) 9 (28.13%)	13 (40.62%) 10 (31.25%) 9 (28.13%)	19 (59.38%) 13 (40.62%)
*miR-124-2 negative* *n* = 38 (54.29%)	29 (76.32%) 9 (23.68%)	16 (42.11%) 13 (34.21%) 9 (23.68%)	6 (15.79%) 32 (84.21%)
*p*-value	0.7856	0.9099	0.0002
*FAM19A4 positive* *n* = 25 (35.71%)	21 (84%) 4 (16%)	12 (48%) 9 (36%) 4 (16%)	19 (76%) 6 (24%)
*FAM19A4 negative* *n* = 45 (64.29%)	31 (68.89%) 14 (31.11%)	16 (35.56%) 15 (33.33%) 14 (31.11%)	13 (28.89%) 32 (71.11%)
*p*-value	0.2541	0.3530	0.0002

Legend: HPV—human papillomavirus.

**Table 5:** Sensitivity analysis of individual miR-124-2 and FAM19A4 genes and combined algorithms for detecting histological categories.

Gene	Histology	Sensitivity	Specificity	PPV	NPV	LR+	LR−	ROC
*miR-124-2*	CIN1	3.1	65.8	7.1	44.6	0.09	1.47	0.25
	CIN2	12.5	71.1	26.7	49.1	0.43	1.23	0.37
	CIN3	25.0	84.2	57.1	57.1	1.58	0.89	0.57
	SCC	28.1	86.8	64.3	58.9	2.14	0.83	0.61
	CIN2+	65.6	42.1	48.8	59.3	1.13	0.82	0.54
	CIN3+	53.1	71.1	60.7	64.3	1.84	0.66	0.62
*FAM19A4*	CIN1	4.0	71.1	7.1	57.1	0.14	1.35	0.38
	CIN2	8.0	71.1	13.3	58.2	0.28	1.29	0.35
	CIN3	36.0	88.9	64.3	71.4	3.24	0.72	0.67
	SCC	36.0	88.9	64.3	71.4	3.24	0.72	0.67
	CIN2+	80.0	48.9	46.5	81.5	1.57	0.41	0.64
	CIN3+	72.0	77.8	64.3	83.3	3.24	0.36	0.73
Combined test	CIN1	5.3	62.5	14.3	35.7	0.14	1.52	0.34
	CIN2	13.2	68.8	33.3	40	0.42	1.26	0.36
	CIN3	28.9	90.6	78.6	51.8	3.09	0.78	0.65
	SCC	26.3	87.5	71.4	50	2.11	0.84	0.60
	CIN2+	68.4	46.9	60.5	55.6	1.29	0.67	0.58
	CIN3+	55.3	78.1	75.0	59.5	2.53	0.57	0.67

Legend: PPV—positive predictive value; NPV—negative predictive value; LR+—positive likelihood ratio; LR−—negative likelihood ratio.

**Table 6:** Multivariable logistic regressions of individual and combined markers for the prediction of histological categories.

Lesion	Model	OR	95% CI Lower Bound	95% CI Upper Bound	*p* Value
CIN1	*miR-124-2*	0.07	0.0085	0.57	0.013
	*FAM19A4*	0.11	0.014	0.94	0.044
	*miR-124-2* adjusted for age	0.07	0.008	0.55	0.012
	*FAM19A4* adjusted for age	0.13	0.015	1.06	0.057
	Combined test	0.11	0.021	0.53	0.006
	Combined test adjusted for age	0.11	0.021	0.54	0.007
CIN2	*miR-124-2*	0.35	0.099	1.24	0.103
	*FAM19A4*	0.21	0.044	1.04	0.056
	*miR-124-2* adjusted for age	0.32	0.088	1.19	0.089
	*FAM19A4* adjusted for age	0.24	0.049	1.21	0.085
	Combined test	0.33	0.10	1.11	0.073
	Combined test adjusted for age	0.33	0.095	1.13	0.078
CIN3	*miR-124-2*	1.78	0.54	5.81	0.341
	*FAM19A4*	4.50	1.31	15.51	0.017
	*miR-124-2* adjusted for age	2.21	0.061	7.97	0.225
	*FAM19A4* adjusted for age	5.51	1.43	21.25	0.013
	Combined test	3.94	0.99	15.65	0.052
	Combined test adjusted for age	3.79	0.91	15.86	0.068
SCC	*miR-124-2*	2.58	0.77	8.71	0.126
	*FAM19A4*	4.50	1.31	15.5	0.017
	*miR-124-2* adjusted for age	3.67	0.55	24.7	0.18
	*FAM19A4* adjusted for age	8.28	0.99	49.3	0.041
	Combined test	2.50	0.70	8.92	0.158
	Combined test adjusted for age	3.28	0.49	21.81	0.219

Legend: HPV—human papillomavirus; NILM—negative for intraepithelial lesion or malignancy; CIN1,2,3—cervical intraepithelial neoplasia 1,2,3; SCC—squamous cell carcinoma; CI: confidence interval; OR: odds ratio.

## Data Availability

The data presented in this study are available on request from the corresponding author. The data are not publicly available due to local policies.

## Abbreviations

The following abbreviations are used in this manuscript:

ASCH	Atypical Squamous Cells That Cannot Exclude High-Grade Squamous Intraepithelial Lesion
ASCUS	Atypical Squamous Cells of Undetermined Significance
BMD	Borderline or Mild Dyskaryosis
CE-IVD	Conformité Européenne In Vitro Diagnostic
CIN1	Cervical Intraepithelial Neoplasia Grade 1
CIN2	Cervical Intraepithelial Neoplasia Grade 2
CIN3	Cervical Intraepithelial Neoplasia Grade 3
DNA	Deoxyribonucleic Acid
ΔΔCt	Delta–Delta Cycle Threshold
ESwab	Specimen Collection Swab (COPAN-Brescia, Italy)
FAM19A4	Family With Sequence Similar 19 Member A4
HGSIL	High-Grade Squamous Intraepithelial Lesion
HPV	Human Papillomavirus
IC	Internal Control
IVD	In Vitro Diagnostic
LGSIL	Low-Grade Squamous Intraepithelial Lesion
LLETZ	Large Loop Excision of the Transformation Zone
lrHPV	Low-Risk Human Papillomavirus
MA, USA	Massachusetts, United States of America
NILM	Negative for Intraepithelial Lesion or Malignancy
ND1000	NanoDrop 1000 Spectrophotometer
Pap test	Papanicolaou Test
PB	Punch Biopsy
PCR	Polymerase Chain Reaction
qMSP	Quantitative Methylation-Specific PCR
RNA	Ribonucleic Acid
SCC	Squamous Cell Carcinoma
USA	United States of America
